# Comprehensive analysis of AHL gene family and their expression under drought stress and ABA treatment in *Populus trichocarpa*

**DOI:** 10.7717/peerj.10932

**Published:** 2021-02-17

**Authors:** Hanzeng Wang, Xue Leng, Jia Yang, Mengqiu Zhang, Minzhen Zeng, Xuemei Xu, Fude Wang, Chenghao Li

**Affiliations:** 1 State Key Laboratory of Tree Genetics and Breeding, Northeast Forestry University, Harbin, China; 2 Library of Northeast Forestry University, Harbin, China; 3 Institute of Forestry Science, Harbin, China

**Keywords:** AT-hook motif containing nuclear localized gene family, Bioinformatic analysis, Transcription factor, qRT-PCR

## Abstract

The AT-hook motif nuclear-localized (AHL) family is a plant transcription factor family, which plays an important role in growth and development and stress responses. We identified and analyzed 37 *AHL* genes in poplar (*Populus trichocarpa*). Phylogenetic analysis classified the *PtrAHL* members into three subfamilies based on their conserved domain. All *PtrAHL* paralogous pairs evolved under purifying selection. The promoter analysis revealed the presence of stress-related and phytohormone-related *cis*-elements of the *PtrAHL* genes. Our analysis of the tissue-specific expression pattern of *PtrAHL* genes indicated their significance in tissue and organ development. Network-based prediction suggested that *PtrAHL* genes may interact with histone deacetylases (HDAC) and participate in the development of organs, such as roots. Drought negatively impacts plant growth and development. ABA is produced under osmotic stress condition, and it takes an important part in the stress response and tolerance of plants. Real-time quantitative PCR (qRT-PCR) showed that *PtrAHL* genes were induced by drought stress and ABA treatment. These insights into the expression of *PtrAHL* genes under stress provide a basis for *PtrAHL* gene functional analysis. Our study will help develop new breeding strategies to improve drought tolerance in poplar.

## Introduction

AT-hook motif nuclear-localized (AHL) family, which serves as transcription factors, exists in all sequenced dicot and monocot terrestrial plants (Zhao et al., 2013). This gene family has two conserved domain, the AT-hook motif and the plant and prokaryote (PPC or Domain of Unknown Function 296, DUF296) domain (Zhao et al., 2013). The AT-hook motif, which contains the core sequence of Arg-Gly-Arg amino acid, followed by Arg-Lys or Pro, binds with the minor groove of AT-rich regions (AA(T/A)T) and changes the architecture of DNA to regulate gene expression (Huth et al., 1997; Xiao et al., 2009). The AT-hook motif vary in the consensus sequence resulting in their divergence into two types (Type-I and Type-II) (Zhao et al., 2013). Type-I motifs possess the Gly-Ser-Lys-Asn-Lys consensus sequence, Type-II motifs contain the Arg-Lys-Tyr at the C-terminus and Arg-Gly-Arg core sequence (Fujimoto et al., 2004). Increasing evidence had showed that AHL proteins contain not only AT-hook motif but also the Plant and Prokaryote Conserved (PPC/DUF296) domain, which contains about 120 amino acids long and exists in prokaryotic proteins (Fujimoto et al., 2004). This domain is essential for nuclear localization and interacts with other transcription factors (Fujimoto et al., 2004). Studies have shown that PPC domain regulates the AHL proteins’ transcriptional activation (Zhao et al., 2013). However, little information is avaliable about this domain’s direct rolein regulating plant growth and development.

AT-hook motif gene family has been characterised in manyplants, including *Arabidopsis thaliana* (Zhao et al., 2013), *Gossypium raimondii* (Zhao et al., 2020), *Oryza sativa* (Kim et al., 2011) and *Zea mays* (Bishop et al., 2020). The AHL genes have been comprehensively analyzed in *Arabidopsis* and are known to regulate plant growth and development. Overexpression of *AtAHL18* promoted root development, longer primary roots and higher lateral root density (Sirl et al., 2020). In *Arabidopsis* roots, *AtAHL3* and its homologous gene, *AtAHL4*, influence the development of vascular tissue boundaries (Zhou, Wang & Lee, 2013). *AtAHL22* binds with the AT-rich sequence in the FLOWERING LOCUS by recruiting histone deacetylases (HDAC) and regulates flowering time (Yun et al., 2012). The SUPPRESSOR OF PHYTOCHROME B4-#3 (*SOB3*) coding an AT-hook protein restricts petiole elongation in *Arabidopsis* (Favero et al., 2020). Besides, several *AHL* genes are known to play crucial roles in abiotic and biotic stresses. *AtAHL20* negatively regulates pathogen-associated molecular patterns (PAPM)-triggered immunity by suppressing PAMP-induced *NHO1* and *FRK1* expression (Lu, Zou & Feng, 2010). AtAHL10 interacts with Highly ABA-Induced 1 (HAI1) protein and participates in low-water potential stress (Wong et al., 2019). In rice, *OsAHL1* positively regulates drought-related genes to enhance resistance at the panicle development stage. Meanwhile, *OsAHL1* overexpression enhanced tolerance to abiotic stresses, such as salt and cold stresses (Zhou et al., 2016). Thus, previous findings confirm that *AHL* genes play crucial roles in plants growth and development as well as biotic and abiotic stress responses by regulating target gene expression or interacting with other proteins.

Abscisic acid (ABA) is one of the most important hormones involved in various plant physiological processes, including growth, development, and stress responses (Lee & Luan, 2012). Drought promotes ABA synthesis in guard cells and induces stomatal closure (Abid et al., 2018). ABA activates gene expression and adaptive physiological changes (Lim et al., 2015). Under the osmotic conditions, ABA stimulates hydrogen peroxide (H_2_O_2_) generation in the guard cells mainly by NADPH oxidase, and the generated H_2_O_2_ molecules mediate ABA-induced stomatal closure by activating plasma membrane calcium channels (Kwak et al., 2003; Li et al., 2017). *OsAHL1* is induecd by 150 µM ABA treatment in rice, while, *OsAHL1* overexpression transgenic lines enhanced drought stress resistance of rice at the seedling stage (Zhou et al., 2016), indicating that *AHLs* may take part in ABA signaling pathways in mediates drought stress response.

Poplar (*Populus trichocarpa*) is a fast-growing, woody plant with economic value and plays an important role in ecosystem sustainability. Poplar genome, wholly sequenced in 2006 (Tuskan et al., 2006), provides a starting point for further molecular biology research. Comprehensive genome-wide analysis based on the sequenced genome will help explore the potential functions of the AHLs. We analyzed the subcellular localization, phylogenetics, gene structure, and conserved motifs, evolutionary relationship, promoter *cis*-elements and protein interaction network of 37 AHLs identified in *P. trichocarpa*. We then analyzed the expression levels of *PtrAHL* genes in the roots and leaves of *P. trichocarpa* under drought stress and ABA treatments. Our findings will provide a basis for further research on the functions of *AHL* s in plants during growth and development and hormone treatment.

## Materials and Methods

### Identification of AHL genes in *P. trichocarpa*

We obtained the amino acid sequences of 29 *AtAHL* proteins (Zhao et al., 2014) from TAIR (The *Arabidopsis* Information Resource; https://www.arabidopsis.org/) database. These sequences were used as a query to blast in Phytozome (version 12.1; https://phytozome.jgi.doe.gov/pz/portal.html), with the following parameters: target type, proteome; program, BLASTP-protein query to protein db; and expect (E) threshold, −1. The redundant sequences were manually detected and deleted. In the candidate *PtrAHL* amino acid sequences, the existence of the conserved AT-hook motif and the PPC domain was confirmed using the SMART database (http://smart.embl-heidelberg.de/) (Letunic & Bork, 2018). The coding amino acid and gene sequences of each *PtrAHL* gene were directly downloaded from Phytozome. The subcellular localization was predicted using WoLF PSORT (https://www.genscript.com/wolf-psort.html) (Horton et al., 2007). The protein length, molecular weight, theoretical pI, aliphatic index and grand average of hydropathy (GRAVY) were identified by using the ProtParam tool on Expasy (https://web.expasy.org/protparam/) (Wilkins et al., 1999).

### Phylogenetic analysis

The amino acid sequences from *O*. *sativa* were obtained from the NCBI database by standard Protein BLAST using the *PtrAHL* genes as query. The Clustal X (version 2.0) and Bioedit (version 7.2.5) were used to perform the multiple sequence alignment with a gap opening penalty and gap extension penalty of 10 and 0.1, respectively. MEGA (version 7.0) was used to analyze the molecular features and phylogenetic relationships of the *AHL* genes of *P. trichocarpa*, *Arabidopsis* and *O*. *sativa* using the maximum likelihood (ML) method with the following parameters: Poisson model; partial deletion (95%); and 500 bootstrap replications (He et al., 2013).

### Gene structure and conserved motif analysis

The exon-intron organization of *PtrAHL* genes was generated using GSDS (Gene Structure Display Server version 2.0: http://gsds.cbi.pku.edu.cn/). MEME (Multiple Em for Motif Elicitation program 5.1.1; http://meme-suite.org/tools/meme) was used to identify the conserved motifs in *PtrAHL* genes using the default parameters and a conserved motif number of 15.

### Calculation of Ka/Ks values

The orthologs of the *PtrAHL* genes in *P. trichocarpa* were identified in DnaSP (DNA Sequence Polymorphism, version 6.12.03). The paralogous genes’ coding sequences were first aligned using Clustal X, and the alignment results and selected protein-coding regions were loaded in DnaSP. Gene pairs originating from duplication events within genome of a single species was regarded as paralogous pairs (Altschul et al., 1997). Finally, the synonymous and nonsynonymous substitutions were selected to calculate the Ka/Ks values for all paralogous genes. The divergence time (T; million years ago, Mya) was calculated as follows: T = Ks/(2 × 6.1 × 10^−9^) Mya (Wei, Xu & Li, 2017).

### Chromosomal location analysis

The chromosomal location of each *PtrAHL* gene in the *P. trichocarpa* genome was confirmed using the PopGenIE (Populus Genome Integrative Explorer, version 3 database (Sjodin et al., 2009). The schematic representation of the homologous chromosomes segments was generated using MapChart (version 2.3). Adobe Illustrator CS6 (Adobe System Incorporated) program was used to construct the physical map based on the information in PopGenIE. Genes separated by ≤5 gene loci in a range of 100 kb were considered as tandem duplicates (Hu et al., 2010).

### Promoter *cis*-element analysis

The promoters of *PtrAHL* genes, 2,000 bp upstream of the translation start site, were obtained from Phytozome. The online database PlantCARE was used to search and locate the *cis*-elements in each promoter (Lescot et al., 2002).

### Plant materials, drought stress and ABA Treatment

*P. trichocarpa* (genotype Nisqually-1) plants were cultured *in vitro* on woody plant medium (WPM) supplied with 20 g/L of sucrose and 5.5 g of agar and maintained in a growth chamber with 16 h/8 h light/dark cycles at 25 °C with a light intensity of 46 µmol photons m^−^^2^ s^−^^1^. For drought stress, three-week-old *in vitro* plants were transferred to WPM medium supplied with 7% PEG6000. For ABA treatment, three-week-old plants were exposed to the WPM medium containing 200 µM ABA. The plants were allowed to grow under these two stress conditions for 0, 3, 6, 12, and 24 h. The untreated plants served as controls. Samples were collected, immediately frozen in liquid nitrogen, and stored in a freezer (−80 °C) for further use. Three independent biological replicates were maintained for each treatment.

### RNA isolation and real-time quantitative PCR (qRT-PCR)

Total RNA of *P. trichocarpa* was extracted using the CTAB (cetyltrimethylammonium bromide) method (Jaakola et al., 2001), and RNA samples were analyzed by agarose gel electrophoresis to ensure integrity. HiScript® II Q Select RT SuperMix for qPCR Kit (Vazyme) was used for reverse transcription, and gDNA Wiper Mix to remove the genome DNA. qRT-PCR was performed using the AceQ® Universal SYBR qPCR Master Mix Kit (Vazyme) following the manufacturer’s instructions. The qRT-PCR was performed on a qTOWER 3G Cycler (Analytik Jena, Germany), and the 2^−ΔΔCT^ method was used to analyze the relative gene expression level. Primer Premier 5 was used to design the primers, and NCBI primer designing tool (https://www.ncbi.nlm.nih.gov/tools/primer-blast/index.cgi?LINK_LOC=BlastHome) was employed to check their specificity. The *P. trichocarpa* actin gene (GenBank ID: XM_002298674) was used as the reference gene (Chu et al., 2014). All *PtrAHL* gene-specific primers used for qRT-PCR are listed in Table S1.

### exHeatmap analysis

The tissue-specific expression values were directly downloaded from the published RNA-seq data (NCBI GEO number GSE6422) in PopGenIE using the accession numbers of each *PtrAHL* (Yang et al., 2008). Heml 1.0.3.7 software was used to visualize the data (Deng et al., 2014) The clustering method selected “Hierarchical” and “Average linkage (default)”. The expression values are listed in the Table S2.

### Gene Ontology (GO) annotation

The functional annotation of *PtrAHL* genes was performed using Blast2GO (version 5.2) as follows: the full-length protein sequences were aligned and mapped using UniProtKB/Swiss-Prot (swissprot_v5). An annotation cutoff: 55, GO weight: 5, and E-value-hit-filte of 1.0E-6 was used for annotating into three GO categories (biological processes, molecular functions, and cellular components).

### Protein interaction network analysis

The STRING (version 11.0; https://string-db.org/cgi/input.pl) database was used to predict the protein interaction network of *PtrAHL* proteins in *P. trichocarpa* (Szklarczyk et al., 2015). The sequence of each protein was used as query, and the “Organism” options *P. trichocarpa* was selected. Then basic settings included “meaning of network edges” set as “evidence” and “active interaction sources” set as “Text-mining, Experiments, Databases, Co-expression, Neighborhood, Gene Fusion and Co-occurrence”. The minimum required interaction score was customized to medium confidence of 0.4. Finally, the protein interaction network was exported and visualized using Cytoscape (version 3.7.2).

### Statistical analysis

Data were analyzed using Student’s t test was performed using SPSS software (version 20, IBM, Chicago, USA) to assess the significant differences between the treatments and control, at *p* <0.05 (*) and *p* < 0.01 (**). The detailed statistical analysis was listed in Table S3.

## Results

### Identification of *AHL* genes in *P. trichocarpa*

We identified 37 *AHL* genes from *P. trichocarpa* whole genome. According to the position on the chromosome, we named these *AHL* genes from *PtrAHL1* to *PtrAHL37*. The protein length ranged from 248 aa to 413 aa, and the molecular weight ranged from 26.2 kDa to 44.2 kDa. Among the different proteins, PtrAHL22 was the largest and *PtrAHL30* was the smallest. The *PtrAHL* proteins showed considerable variations in the theoretical pI values (5.05 to 10.11). PtrAHL25 had the lowest GRAVY score, while PtrAHL16 had the highest (Table 1). WoLF PSORT predicted nuclear localization for 23 proteins, cytoplasmic localization for 18.9% and chloroplast localization for four proteins. PtrAHL3/34 and PtrAHL8/9 were predicted in the plasma membrane and endoplasmic reticulum, respectively. The detailed information is provided in Table 1.

**Table 1:** Basic information about AHLs in *P. trichocarpa*.

**Name**	**gene ID**	**Protein Length (a.a.)**	**Molecular weight (Da)**	**Theoretical pI**	**GRAVY**	**The predicted location of PtrAHL proteins**
PtrAHL1	Potri.001G104900	347	36451.89	9.49	−0.399	Nuclear
PtrAHL2	Potri.001G115200	302	32372.13	6.45	−0.588	Nuclear
PtrAHL3	Potri.001G115800	336	34263.7	9.61	−0.326	Plas
PtrAHL4	Potri.001G142800	298	30363.74	6.33	−0.24	Cytoplasmic
PtrAHL5	Potri.001G143500	377	38546.18	9.84	−0.492	Nuclear
PtrAHL6	Potri.001G275700	302	31683.35	7.19	−0.279	Cytoplasmic
PtrAHL7	Potri.002G003800	302	30549.85	8.82	−0.32	Nuclear
PtrAHL8	Potri.002G005000	382	38038.76	9.85	−0.174	E.R._plas
PtrAHL9	Potri.002G059400	266	28117.27	9.43	−0.125	E.R._plas
PtrAHL10	Potri.002G105000	305	32029.7	7.18	−0.488	Nuclear
PtrAHL11	Potri.002G148600	325	33831.09	9.1	−0.33	Nuclear
PtrAHL12	Potri.002G149300	298	31538.28	7.14	−0.496	Nuclear
PtrAHL13	Potri.002G158200	328	34050.48	9.54	−0.22	Chloroplast
PtrAHL14	Potri.003G090900	375	38359.67	9.69	−0.5	Nuclear
PtrAHL15	Potri.003G091300	298	30524.14	5.88	−0.2	Cytoplasmic
PtrAHL16	Potri.003G116500	269	28186.53	9.92	−0.064	Chloroplast
PtrAHL17	Potri.003G116900	303	32546.33	6.45	−0.598	Nuclear
PtrAHL18	Potri.003G126500	346	36135.46	9.78	−0.439	Nuclear
PtrAHL19	Potri.004G189200	344	35412.55	8.45	−0.366	Nuclear
PtrAHL20	Potri.004G208600	297	29833.13	7.95	−0.288	Nuclear
PtrAHL21	Potri.005G156700	306	32049.71	6.8	−0.464	Nuclear
PtrAHL22	Potri.005G202700	248	26283.19	9.95	−0.179	Cytoplasmic
PtrAHL23	Potri.005G256500	399	39795.52	9.74	−0.273	Nuclear
PtrAHL24	Potri.005G257200	301	30462.66	9.35	−0.376	Nuclear
PtrAHL25	Potri.008G058700	336	35396.93	5.93	−0.626	Nuclear
PtrAHL26	Potri.008G164500	298	30854.13	5.05	−0.455	Nuclear
PtrAHL27	Potri.009G070300	301	30973.7	7.79	−0.212	Cytoplasmic
PtrAHL28	Potri.009G150000	349	35836.20	9.36	−0.326	Nuclear
PtrAHL29	Potri.010G074200	295	30400.78	5.75	−0.348	Cytoplasmic
PtrAHL30	Potri.010G200100	413	44159.13	6.42	−0.509	Nuclear
PtrAHL31	Potri.012G129500	350	36517.06	9.19	−0.412	Chloroplast
PtrAHL32	Potri.013G044000	369	38176.29	8.66	−0.517	Nuclear
PtrAHL33	Potri.013G044500	284	29372.71	5.48	−0.482	Nuclear
PtrAHL34	Potri.014G070000	324	33699.04	9.1	−0.342	Plas
PtrAHL35	Potri.014G070800	300	31927.65	6.5	−0.534	Nuclear
PtrAHL36	Potri.014G082100	336	34760.23	10.11	−0.27	Chloroplast
PtrAHL37	Potri.019G014200	365	37890.06	9.61	−0.488	Nuclear

### Phylogenetic analysis of *PtrAHL* genes

**Figure 1:**
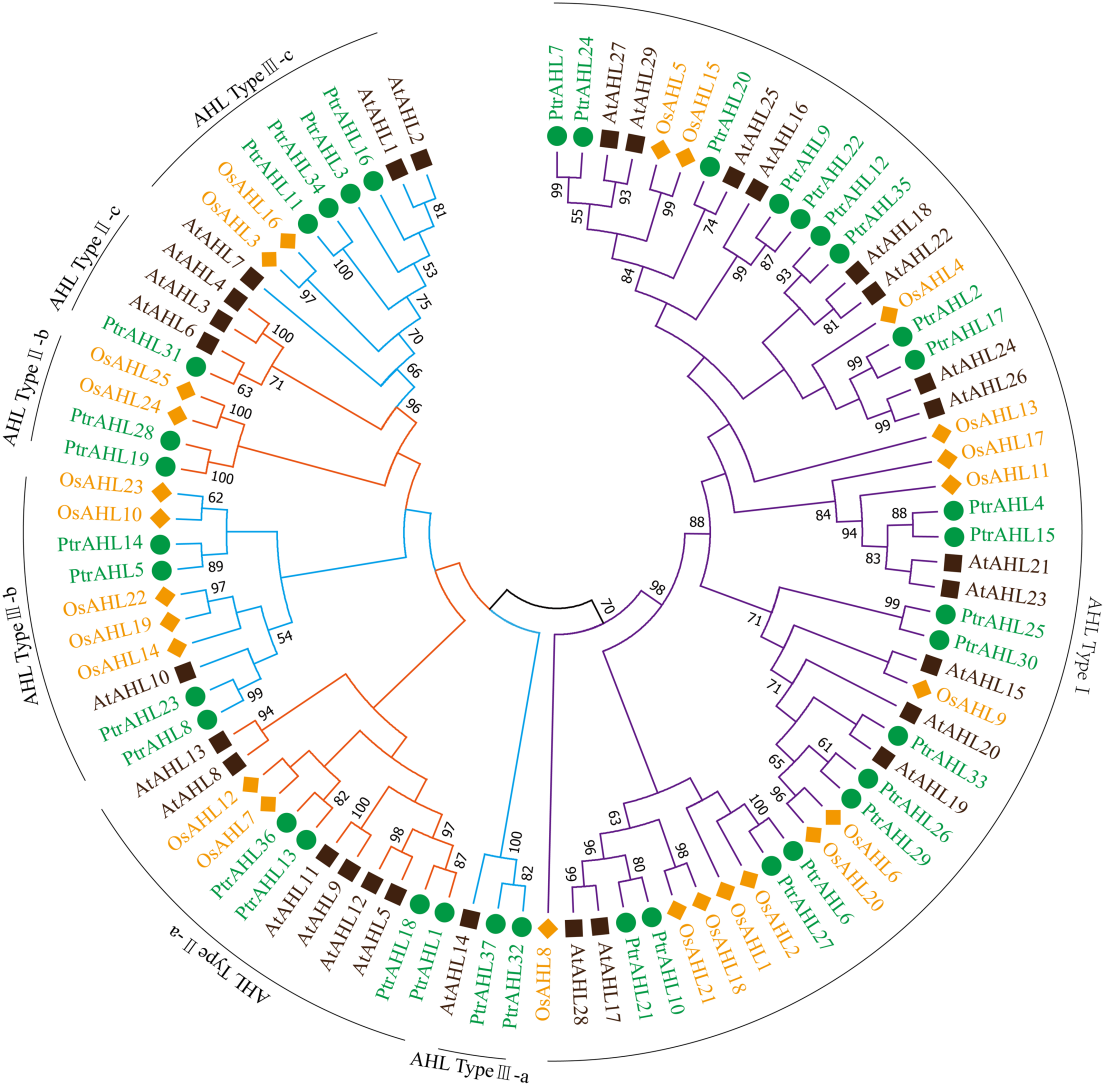
Phylogenetic analysis of the AHL gene family members from *P. trichocarpa*, *O. sativa* and *Arabidopsis*. Branches with less than 50% bootstrap support were collapsed. Different colors represent different species. Seven distinct clades were marked as AHL Type-I, IIa-c and IIIa-c. The phylogenetic tree was constructed using the Maximum Likelihood (ML) method of MEGA 7.0 with 500 bootstrap replicates.

The full-length amino acid sequences of 37 *AHLs* from *P. trichocarpa*, 29 from *Arabidopsis* and 25 from *O. sativa* were used to construct an unrooted tree following the maximum likelihood (ML) method to analyze the evolutionary relationship. MEGA 7.0 program was used to visualize the results. Seven distinct clades were identified (Fig. 1; Bishop et al., 2020) named AHL Type I, AHL Type II a-c and AHL Type III a-c. Type-I possessed RGRPAGSKNKPKP and RGRPPGSKNKPKP conserved sequences and the PPC domain; Type II a-c contained two AT-hook motifs (RGRPRKY and RGRP conserved sequences) and the PPC domain; Type III a-c contained RGRPRKY sequence and the PPC domain. AHL Type-I had the largest number of members, with about 59.45% of *PtrAHL* genes, while AHL Type II a-c and Type III a-c had had 21.62% and 18.93%, respectively. Otherwise, PtrAHL1, 3, 4, 7, 8, 9, 10, 15, 16, 21, 22, 24, 31, 32, 33, 35 and 37 clustered with the *Arabidopsis* AHL protein. These foundings indicate that the PtrAHL proteins are more closely related to those of *Arabidopsis* than of *O. sativa*. The detailed phylogenetic distance is provided in Table S4.

### Phylogenetic analysis, gene structure and conserved motifs

**Figure 2:**
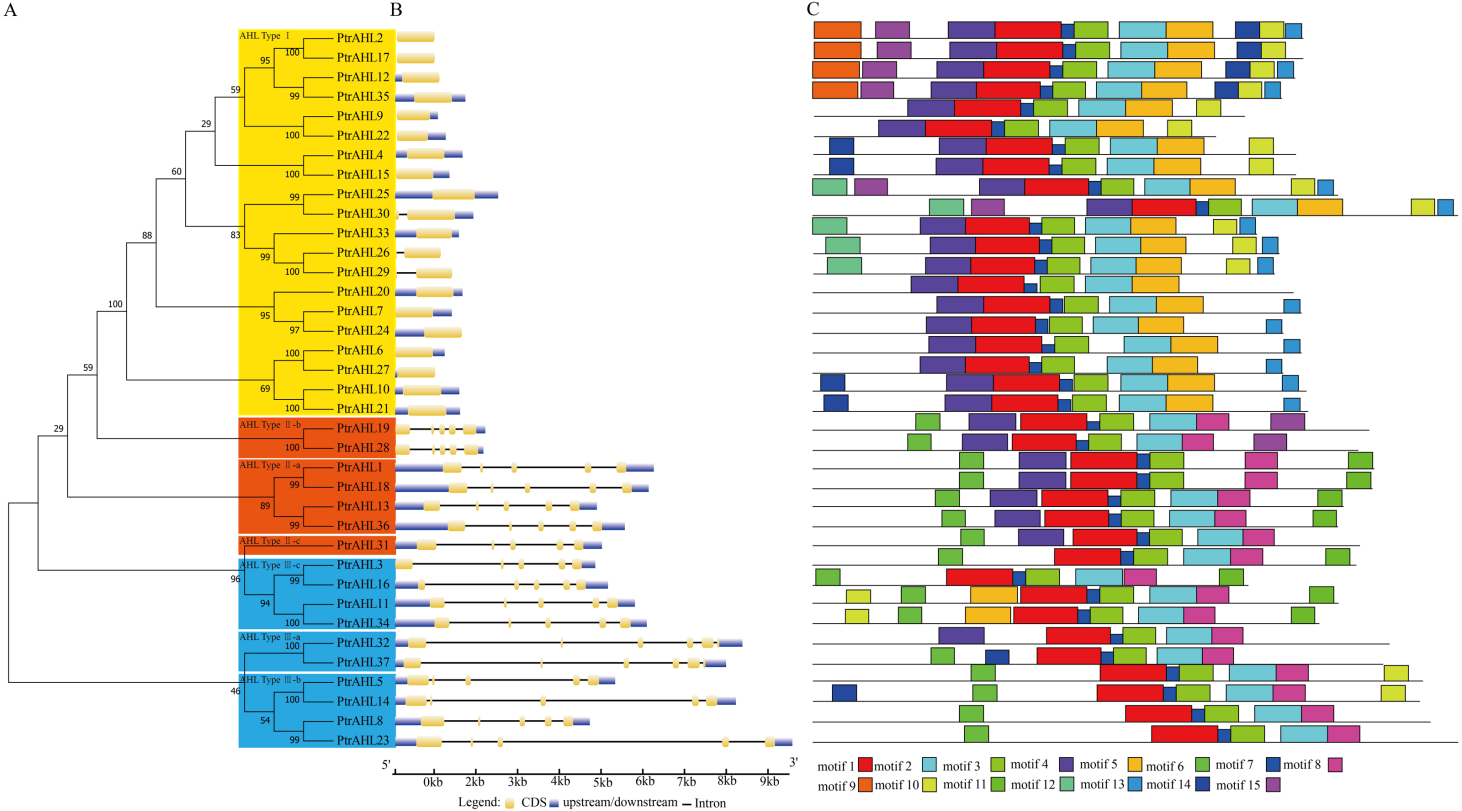
Phylogenetic relationships, gene structure and conserved motifs of AHLs in *P. trichocarpa*. (A) Multiple alignment of full-length amino acid sequences of *PtrAHL* genes was carried out with ClustalX 2.0. The phylogenetic tree was constructed using Maximum Likelihood method with MEGA7.0. Yellow background represents the AHL Type-I subfamily, Orange background represents the AHL Type-IIa-c subfamily and Blue background represents the AHL Type-IIIa-c subfamily. (B) Exon/intron structures of *PtrAHL* gene family. (C) The conserved motifs were obtained from MEME website. Fifteen different kinds of conserved motifs were marked with different colors.

We constructed an phylogenetic tree to investigate the relationships among *PtrAHLs* using their corresponding protein sequences by MEGA 7.0. We identified 16 paralogous pairs with strong bootstrap support (90%; Fig. 2A). We analyzed the exon-intron organization using the full-length coding sequence of AHLs with their corresponding genomic DNA sequences in *P. trichocarpa*. Most *PtrAHL* genes of the same groups had similar exon-intron lengths and numbers, especially the paralogous pairs within the same group (Fig. 2B). *PtrAHL1* and *PtrAHL18* belonging to AHL Type-II a, possessed four introns of similar lengths. Both *PtrAHL9* and *PtrAHL22*, of AHL Type-I subfamily, contained coding sequences and downstream, with no introns.

We further used MEME to confirm the conserved motifs in PtrAHL proteins. Fifteen conserved motifs, named motif 1 to motif 15, were found (Fig. 2C). All PtrAHLs contained motif 1, the AT-hook motif. The paralogous pairs of PtrAHLs possessed similar motifs. Interestingly, PtrAHL14 had one more motif (motif 15) than its homologous gene, PtrAHL5. PtrAHL32 had one more motif (motif 4) than its homolog PtrAHL37, however, PtrAHL37 possessed an additional motif 6 than PtrAHL32, Notably, PtrAHL31 had RGRPRKY and RGRPLESVKKQHN conserved motifs; however, it was classified into the AHL Type-I subfamily, indicating similarity with PtrAHL3/16 and PtrAHL11/34. These observations suggest functional diversity among the AHL proteins. Detailed information about motifs is listed in Table S5.

### Chromosomal locations and gene duplication

**Figure 3:**
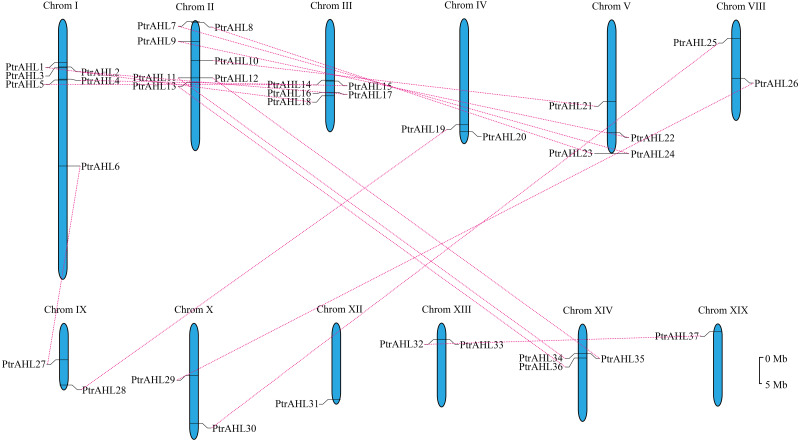
The chromosome locations and segmental paralogous pairs of *PtrAHL* gene family members. The *PtrAHL* genes distributed in 12 chromosomes in *P. trichocarpa* genome was listed. The paralogous pairs were connecting with a red dotted line. The scale bar is 5 Mb.

We mapped on 19 linkage groups (LGs) to determine the distribution of *PtrAHL* genes. Six *PtrAHL* genes (*PtrAHL7, 8, 9, 10, 11,* and *12*) were located on LG II, followed by another six (*PtrAHL1, 2, 3, 4, 5,* and *6*) on LGI (Fig. 3). Five *AHL* genes were detected on LG III and four were located on LG V. *PtrAHL34, 35,* and *36* were located on LG XIV. Besides, LG IV, VIII, IX, X, and XIII each contained two *PtrAHL* genes. *PtrAHL 31* and *PtrAHL37* were located on LG XII and XIX, respectively. No genes were detected on the other LGs.

We further calculated the Ka/Ks values using DnaSP to better understand the evolutionary constraints associated with the *PtrAHL* genes. The Ka/Ks value was less than 1 for almost all *PtrAHL* genes, except for *PtrAHL7* and *PtrAHL24* (Table S5). indicating the evolution of the majority of paralogous pairs was under purifying selection. The Ka/Ks value of *PtrAHL7*/*PtrAHL24* was 1.4901, suggesting the influence of positive selection. The divergent date of these 16 paralogous pairs was estimated to have occurred between 1.36 to 58.62 Mya (Table S6).

### Promoter *cis*-element analysis

**Figure 4:**
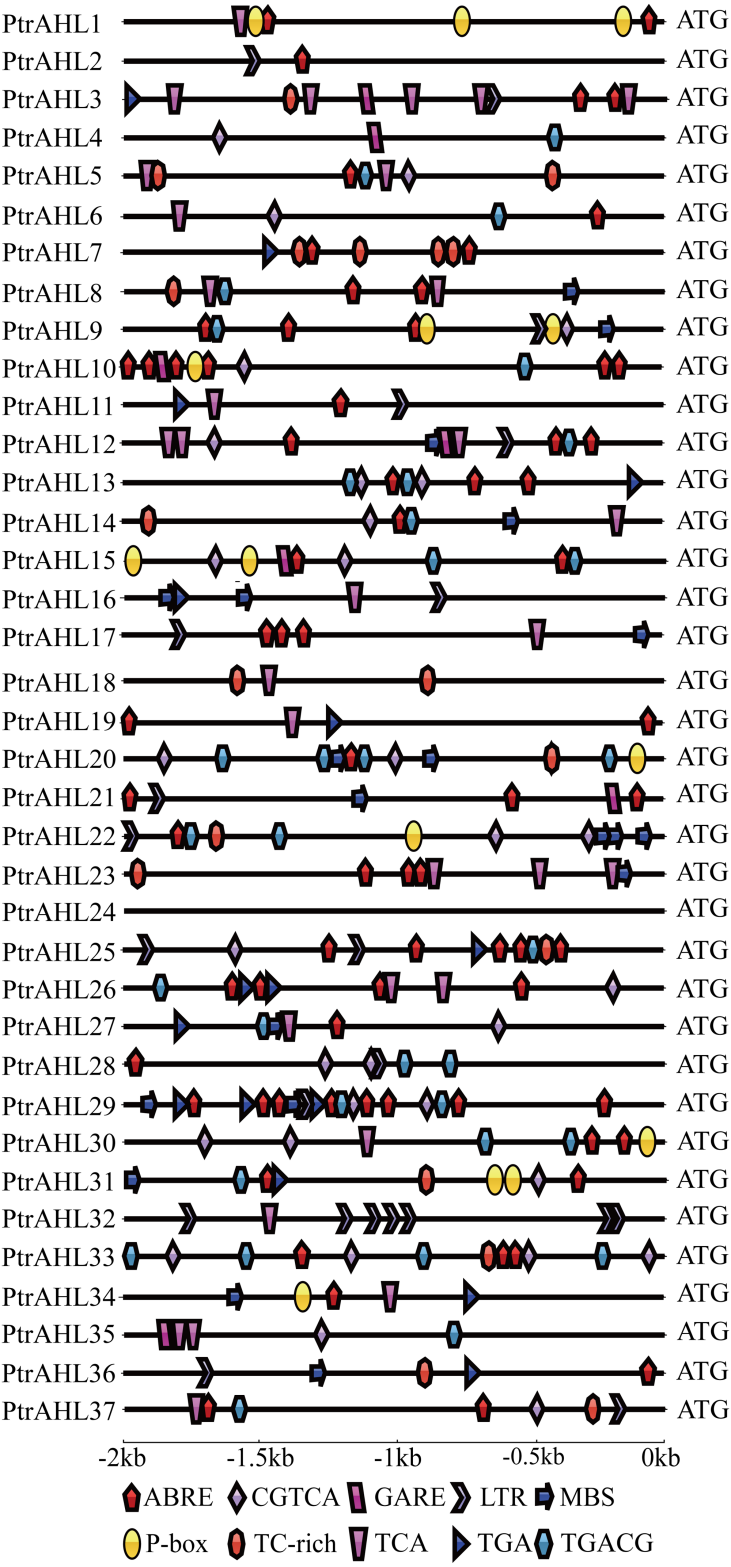
Abiotic- and phytohormone-related *cis*-elements in *PtrAHL* gene promoters. The IBS: Illustrator for Biological Sequences and Adobe Illustrator CS6 software were used to visualize the location of cis-elements in each PtrAHL gene. Different shapes represent different *cis*-elements.

We analyzed the putative stress-related and phytohormone-related *cis*-elements in the promoter region of *PtrAHL* genes using PlantCARE to understand these genes’ possible regulatory mechanisms after phytohormone treatment and under abiotic stress.. Ten different phytohormone-related and stress-related *cis*-elements were identified (Fig. 4). All genes, except *PtrAHL4, 16, 18, 24, 32,* and *35* contained the ABA-responsive element (ABRE). *PtrAHL29* possessed eight ABRE. Besides, *PtrAHL21* and *22* contained methyl jasmonate (MeJA)-responsive element (CGTCA/TGACG), and 20 other *PtrAHLs* possessed salicylic acid-responsive TCA element. *Ptr3, 4, 10, 12, 15, 21,* and *35* contained one GARE-motif, which acted as gibberellin-responsive element. Further, 15 *PtrAHL* genes possessed TGA (auxin-responsive element), 9 members possessed P-box (gibberellin-responsiveness element), and 14 contained TC-rich repeats (defense- and stress- responsive element). Meanwhile, 15 *PtrAHL* genes possessed abiotic stress-related *cis*-elements, such as LTR (low-temperature- responsive element) and MBS (MYB binding site, drought-inducible element). Seven LTR *cis*-elements were found *PtrAHL32*. However, we failed to detect any phytohormone-related or stress-related *cis*-elements in *PtrAHL24*.

### Gene Ontology (GO) annotation

**Figure 5:**
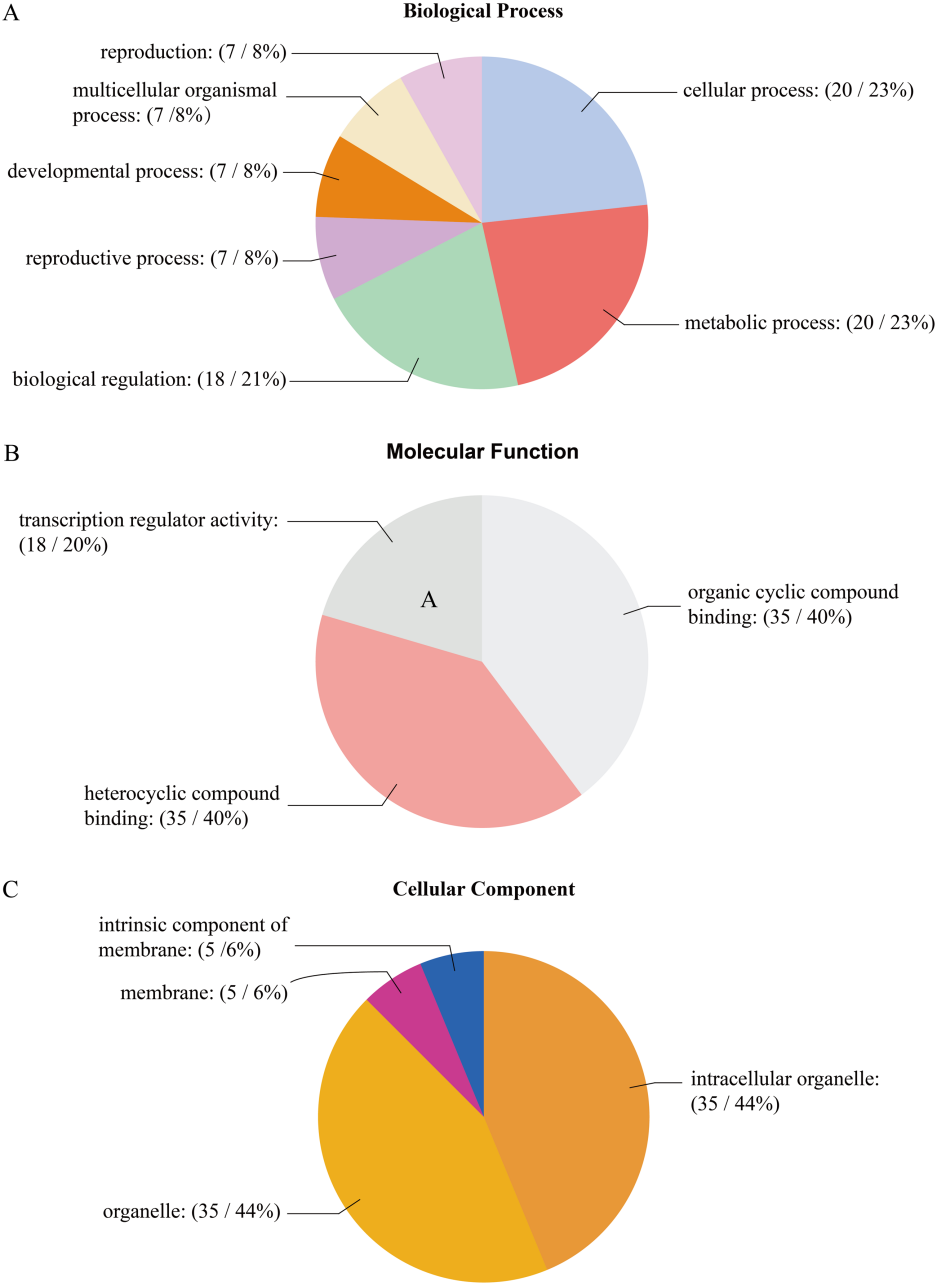
The gene ontology analysis of *PtrAHL* gene family. The GO analysis of *PtrAHL* genes predicted for their involvements in (A) biological processes, (B) molecular functions, and (C) cellular components.

The GO analysis of *PtrAHL* genes revealed their roles in multiple biological processes (Fig. 5). Cellular process and metabolic process had the largest number of *PtrAHL* genes, followed by regulation of biological process and biological regulation. Approximately 6% of the *AHL* genes were related to reproduction, multicellular organismal process, developmental process, and reproductive process. For molecular function, 14 *PtrAHL* genes were functionally enriched in heterocyclic compound binding and organic cyclic compound binding. Besides, 21% of *PtrAHL* genes were involved in transcription regulator activity. Cellular component prediction showed that 47% of *PtrAHL* genes were located in intracellular membrane-bound organelles and membrane-bounded organelles and two were integral components of membrane. The detailed information about GO annotation is listed in Table S7.

### Expression profile of *PtrAHL* genes in different tissues or organs

**Figure 6:**
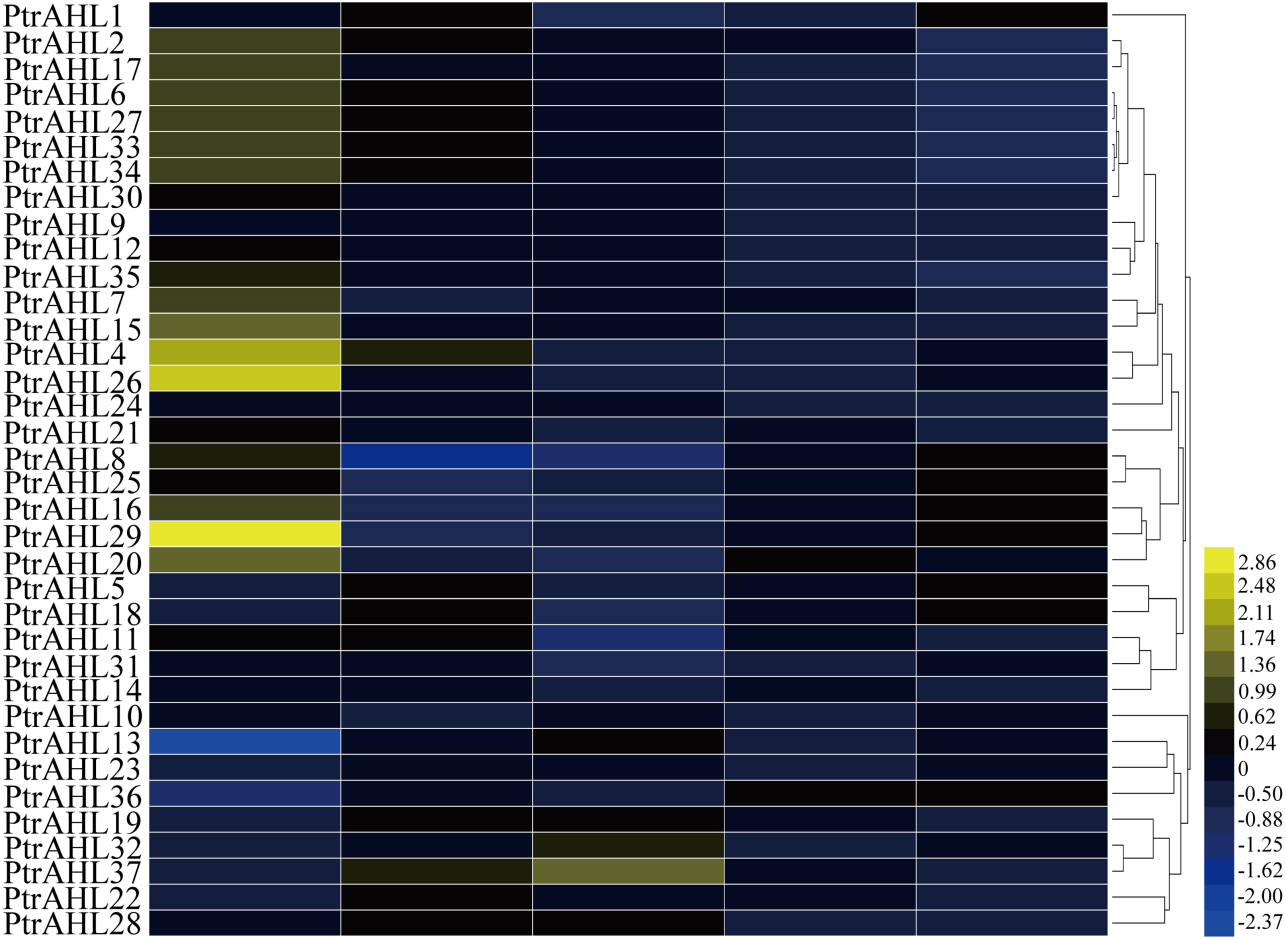
The tissue specific expression pattern of *PtrAHL* genes in different organs. Heatmap shows hierarchical clustering of the AHL gene expression across five various tissues including roots, young leaves, mature leaves, internodes and nodes. The data were obtained from PopGenIE v3 (Yang et al., 2008). The color scale represents the values of log_2_ fold change, yellow represents high level and blue indicates low level of transcript abundances.

To further investigate the function of *PtrAHL* genes in the development, we downloaded the available microarray datasets (accession number: GSE6422) (Yang et al., 2008) to generate a heatmap based on the tissue-specific expression in *P. trichocarpa*. We obtained the expression profiles of 36 *PtrAHL* genes. however, *PtrAHL3* showed no expression in the selected dataset. The *PtrAHL* genes demonstrated different expression patterns across the various developmental stages of tissues or organs in *P. trichocarpa* (Fig. 6). Twenty-four *PtrAHL* genes showed high expression in roots; 64.86% exhibited high expression in young leaves, whereas most of the *PtrAHL* genes showed low expression in mature leaves. *PtrAHL11* and *PtrAHL14* showed high expression in both roots and young leaves, low expression in mature leaves and nodes, and no expression level in internodes. A similar expression profile was observed for *PtrAHL24* and *PtrAHL27* as well as *PtrAHL33* and *PtrAHL34*. Notably, *PtrAHL4, 26* and *29* showed high expression in root. Most of the *PtrAHL* genes showed low or no expression in internodes and nodes.

### Expression pattern of *PtrAHL* genes under drought stress

**Figure 7:**
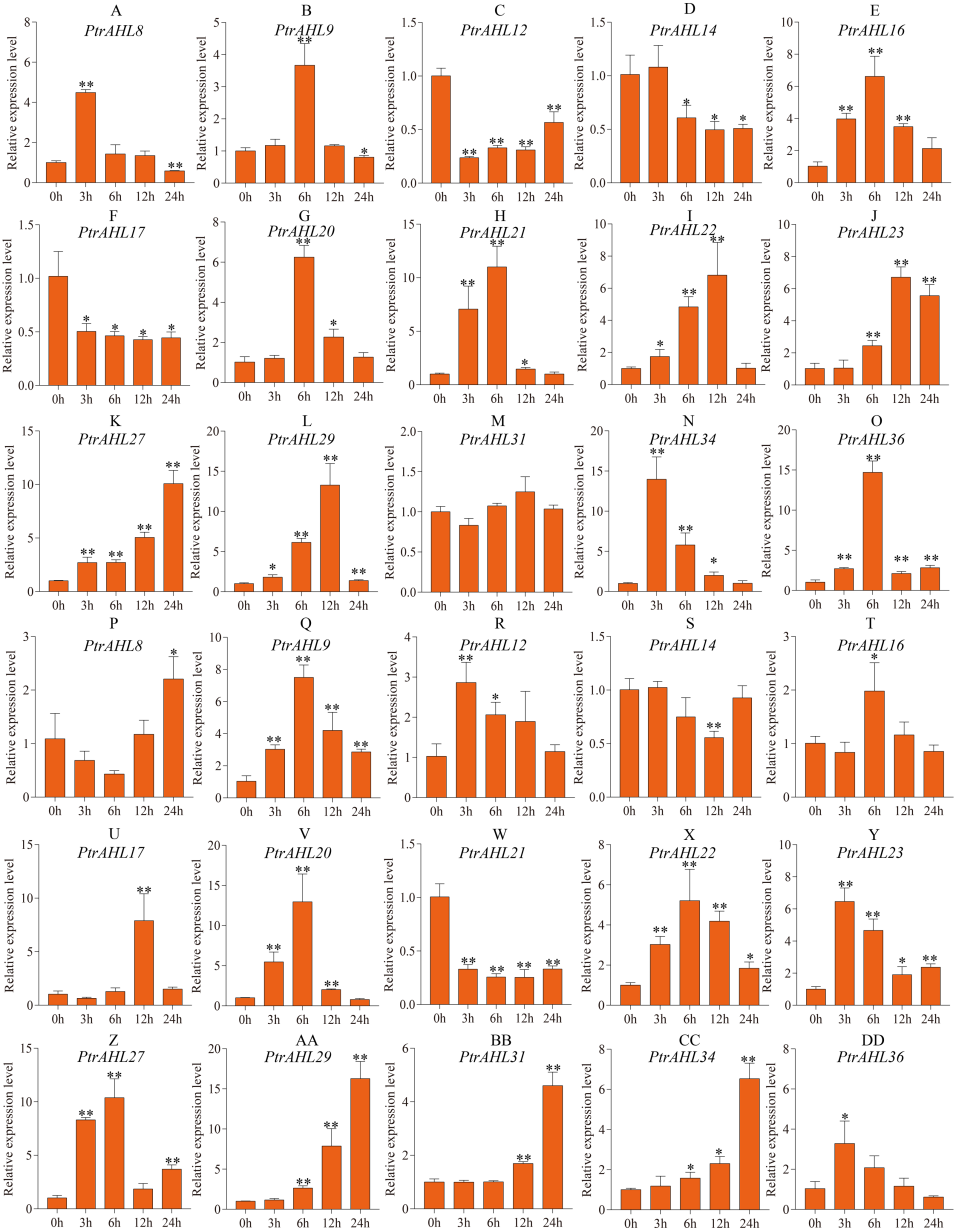
The relative expression level of 15 selected *PtrAHL* genes under drought stress by qRT-PCR. (A-O) The expression analysis of 15 selected *PtrAHL* genes in roots under drought stress. (P-DD) The expression pattern of 15 selected PtrAHL genes in leaves under drought stress. Error bars represent the deviations from three biological replicates. The *x*-axis represents the time points after drought stress. Asterisks indicate the expression level of drought stress groups show a significant difference in transcript abundance comparing with the control (0h) (* *p* < 0.05, ** *p* < 0.01).

Previous studies have shown that MBS *cis*-element helps the plant cope with drought stress (Xu et al., 2019). Our analysis of *cis*-elements predicted MBS *cis*-element in 15 *PtrAHL* genes (Fig. 4). We, therefore analyzed the relative expression levels of these 15 *PtrAHL* genes in both roots and leaves of *P. trichocarpa* under drought stress. In roots, 86.67% of *PtrAHL* genes were induced under drought stress (Fig. 7). *PtrAHL27*, *29,* and *36* were significantly up-regulated at all time points (*p* < 0.01), while, *PtrAHL12*, *14,* and *PtrAHL17* were remarkably down-regulated *PtrAHL34* was rapidly induced by drought stress at 6 h, with 15-fold expression and gradually declined to normal level at 24 h. *PtrAHL31* showed no significant change in expression in roots compared with the untreated control. In leaves, all genes but *PtrAHL14* and *PtrAHL21,* were induced by drought stress. *PtrAHL14* was downregulated at 12 h, and *PtrAHL21* was suppressed at all time points. *PtrAHL16* and *PtrAHL17* were only upregulated at 6 h and 12 h, respectively. *PtrAHL31* showed no significantly early expression (3 h and 6 h) but was upregulated at 12 h and 24 h compared with the control, suggesting late response. Meanwhile, *PtrAHL12* showed the opposite trend. *PtrAHL20* and *PtrAHL22* showed similar expression patterns in both roots and leaves; ohey were upregulated at 3 h and eaked at 6 h) and then declined at 12 h and 24 h (Fig. 7).

### Expression pattern of *PtrAHL* genes under ABA treatment

**Figure 8:**
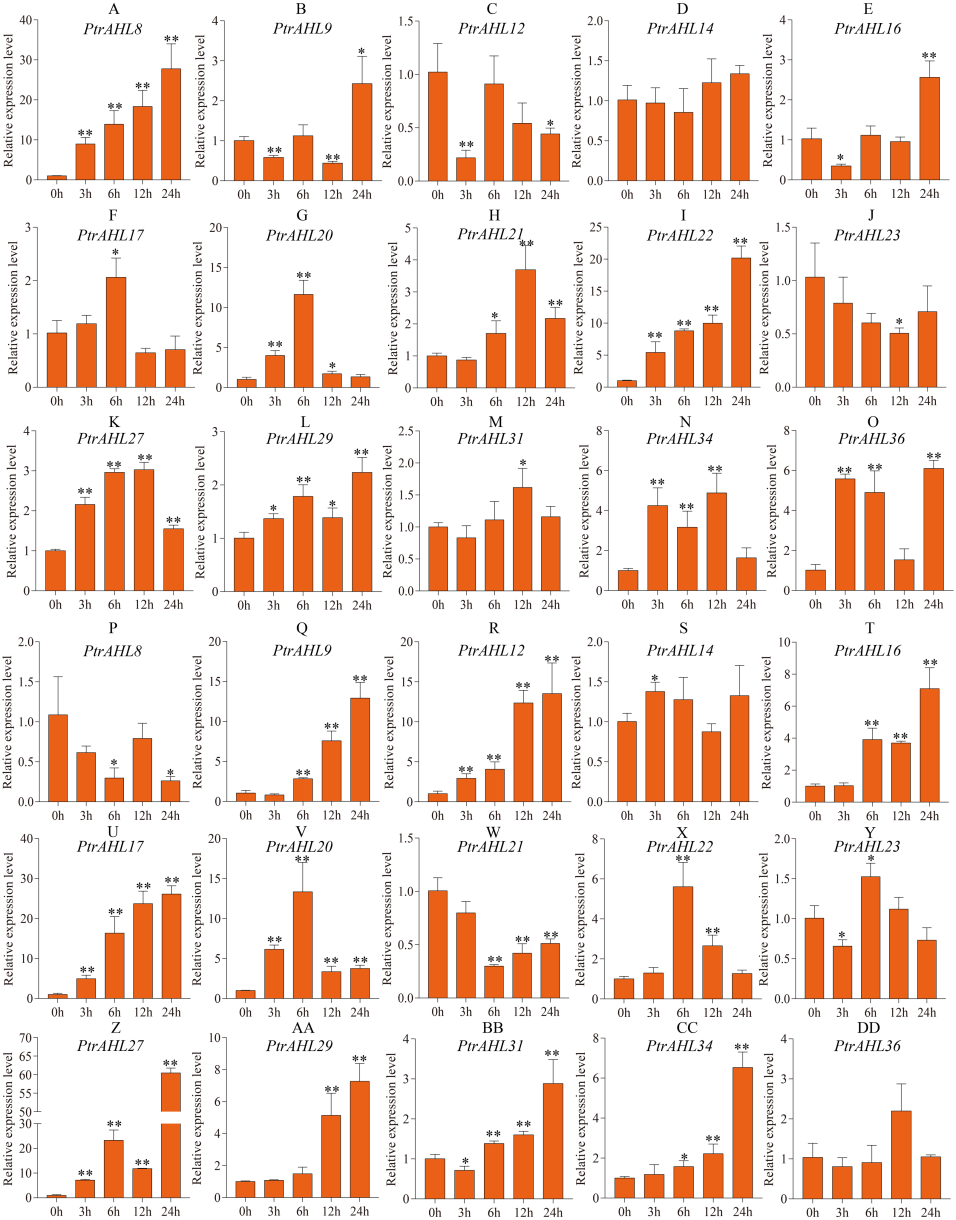
The relative expression level of 15 selected *PtrAHL* genes under ABA treatment by qRT-PCR. (A-O) The expression analysis of 15 selected *PtrAHL* genes in roots under ABA treatment. (P-DD) The expression pattern of 15 selected *PtrAHL* genes in leaves under ABA treatment. Error bars represent the deviations from three biological replicates. The *x*-axis represents the time points after 100mM ABA treatment. Asterisks indicate the expression level of ABA treatment groups show a significant difference in transcript abundance comparing with the control (0h) (* *p* < 0.05, ** *p* < 0.01).

To better understand *PtrAHL* genes’ role in response to ABA treatment, we analyzed the expression levels of 15 *PtrAHL* genes in both roots and leaves of *P. trichocarpa* under 200 µM ABA treatment at 3, 6, 12, and 24 h. In roots, *PtrAHL8, 9, 16, 17, 20, 21, 22, 27, 29, 31, 34,* and *36* were induced by ABA treatment; *PtrAHL12* and *PtrAHL23* were suppressed, *PtrAHL14* showed no significant change in expression compared with the control (Fig. 8). *PtrAHL8* and *PtrAHL22* were significantly upregulated at the initial time points and peaked at 24 h (*p* < 0.01), with 30-fold and 20-fold expression levels compared with control, respectively. *PtrAHL27* and *29* were remarkably upregulated at all time points. *PtrAHL9*, *12,* and *16* were downregulated at 3 h. *PtrAHL17* and *PtrAHL31* were significantly upregulated at 6 h and 12 h (*p* < 0.05), respectively. *PtrAHL20* was significantly upregulated during early response and elevated to a level comparable with that of the untreated control at 24 h. In leaves, 12/15 *PtrAHL* genes were induced, two were suppressed, and only one displayed no change. *PtrAHL12*, *17*, *20,* and *27* were dramatically upregulated at all time points (*p* < 0.01), *PtrAHL12* and *PtrAHL17* were gradually upregulated at 3, 6 and 12 h and peaked at 24 h. with 13-fold and 25-fold expression, respectively. The expression level of *PtrAHL27* was about 60-fold at 24 h. *PtrAHL21* was significantly downregulated (*p* < 0.01), whereas, *PtrAHL9* and *PtrAHL16* were remarkably upregulated at 6, 12, and 24 h (*p* < 0.01) (Fig. 8). *PtrAHL16, 17, 20, 22, 27, 29, 31,* and *34* were induced in both roots and leaves by ABA treatment (Fig. 8).

### Analysis of the AHL protein interaction network in *P. trichocarpa*

**Figure 9:**
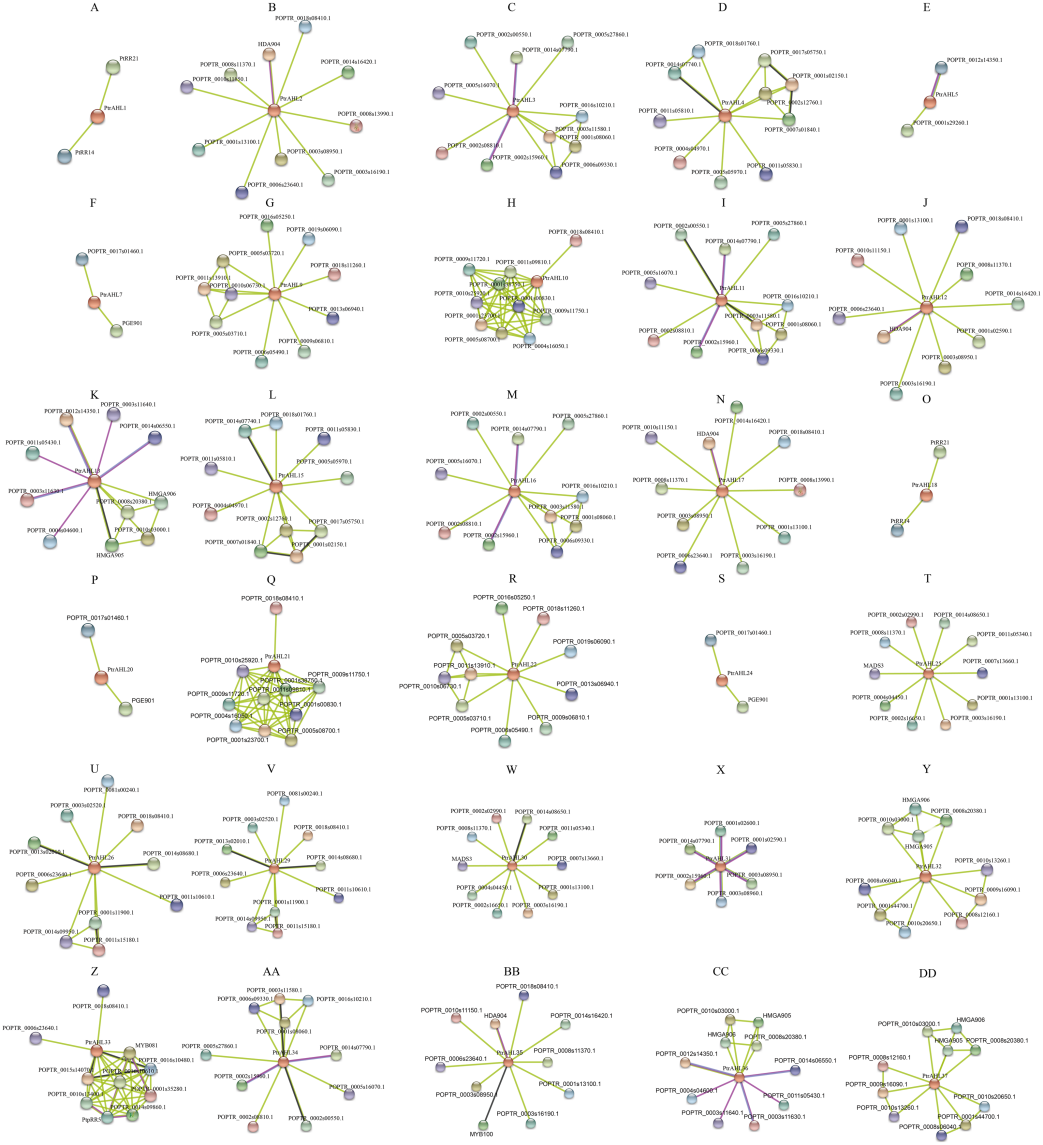
The predicted protein interaction network of PtrAHL proteins. (A-DD) The potential protein interaction networks of each PtrAHL protein were predicted by STRING database. Different colored lines represent different evidence of interaction.

We used the protein sequences of *P. trichocarpa* AHLs to predict the protein interaction network using STRING, We totally obtained 27 protein interaction networks, excluding PtrAHL6, 8, 14, 19, 23, 27, and 28 (Fig. 9), indicating the interaction of PtrAHL proteins interact with other proteins in response to phytohormone treatment and environmental stress as well as during growth and development in *P. trichocarpa*. Our analysis predicted an interaction between PtrAHL2 and *POPTR_0018s08410*, the homologous gene of *AtCPK4*,that regulates the calcium-mediated ABA signaling pathway (Zhu et al., 2007). We found that the homologous proteins, including PtrAHL1 and PtrAHL18, PtrAHL2 and PtrAHL17, PtrAHL3 and PtrAHL16, PtrAHL4 and PtrAHL15, PtrAHL7 and PtrAHL24, PtrAHL9 and PtrAHL22, PtrAHL10 and PtrAHL21, PtrAHL12 and PtrAHL35, PtrAHL25 and PtrAHL30,and PtrAHL26 and PtrAHL29, shared similar protein interaction networks, indicating a conserved protein interaction domain in each protein pair.

## Discussion

### The AHL gene family in *Populus*

AHL family includes transcription factors with one or two AT-hook motifs and a PPC/DUF296 domain (Zhao et al., 2013). In this study, we identified 37 *PtrAHL* genes in *P. trichocarpa* using 29 *Arabidopsis* AHL protein sequences. A previous study identified 29 members in *P. trichocarpa* (Zhao et al., 2014). Here, we reveal another nine AHL genes (*PtrAHL3, 5, 6, 14, 16, 23, 26, 28* and *34*).

Our analysis of predicted localization of *PtrAHL8* and its homologous gene, *PtrAHL23* were in the endoplasmic reticulum and nucleus, respectively. Furthermore, the homologs, PtrAHL3/16, PtrAHL9/22, PtrAHL11/34, and PtrAHL26/29 did not have the same subcellular location, indicating that homologs may also differ in their functions and signal transduction. Studies have identified that AHL proteins localize to the nucleus, while our analysis predicted the localization of 14 PtrAHL proteins in the the plasma membrane, cytoplasm, endoplasmic reticulum, and chloroplast. Prior research has demonstrated that both the AT-hook motif and PPC/DUF296 domain contribute to nuclear localization (Sgarra et al., 2006; Street et al., 2008). Therefore, we speculate the that the presence of other sequences that influence the localization. AHL1 is mainly localized in the nucleoplasm, little in the nucleolus region (Fujimoto et al., 2004). Therefore, a lot of work is needed to verify this.

### The promoter *cis*-elements of AHLs in *Populus*

Under biotic and abiotic stresses, promoter *cis*-elements such as ABRE, CGTCA, GARE-motif, TGA-element, P-box, LTR, TCA-element, TC-rich repeats, TGACG motif and MBS *cis*-element play pivotal roles in the transcriptional regulation in plants (Wang et al., 2020). We identified at least one phytohormone-related or stress-related *cis*-element in all *PtrAHL* family members, except for *PtrAHL24*, indicating their role in regulating the response.We also found the LTR and MBS *cis*-elements in many *PtrAHL* promoters, including *PtrAHL9, 12, 16, 17, 22, 23, 29,* and *36*, indicating their role under low temperature and drought stress. These findings suggest that *AHLs* may play an important role in response to abiotic stress and phytohormone treatment in *P. trichocarpa*.

### Transcript profiles of AHL genes under drought stress and ABA treatment in *Populus*

Plants require water to maintain normal physiological processes, including growth, development, and reproduction (Fang & Xiong, 2015). To overcome and survive drought, woody plants have developed several strategies, including reprogramming gene expression (Debnath, Pandey & Bisen, 2011). Besides, *cis*-elements, especially the MBS *cis*-element, play an important role in drought stress. We identified 15 *PtrAHL* genes with the MBS *cis*-element. In plants, roots are the first organs to perceive drought stress (Lynch, 1995), Moreover, drought influences various events in leaves, including stomatal closure to prevent water evaporation (Qin et al., 2020). Therefore, we analyzed the expression levels of these 15 *PtrAHL* genes in roots and leaves of *P. trichocarpa* under drought stress. *PtrAHL8, 9, 16, 20, 22, 23, 27, 29, 34,* and *36* were induced by drought stress in both roots and leaves, indicating their role in drought response. Interestingly, *PtrAHL12* was induced by drought in leaves but significantly suppressed in roots at all time points. Meanwhile, the expression level of *PtrAHL31* was higher than the control at 12 h and 24 h in leaves, with no change in expression in the roots, These results indicate the tissue-specific expression of the genes. *OsAHL1* (*LOC_Os11g05160*), the homologous gene of *PtrAHL34*, enhanced drought tolerance by directly regulating its target genes (*HSP101, OsCDPK7, OsRNS4, Rab16b and AP2-EREBP*) (Zhou et al., 2016). *OsAHL1* also participates in root development under drought (Zhou et al., 2016). In our experiments, *PtrAHL34* was strongly induced by drought in both roots and leaves, suggesting that similar functions for the homologous genes in different species.

Studies have shown that ABA is the most critical abiotic stress-related hormones and is involved in various physiological processes, including growth, development, and reproduction (Dong, Park & Wang, 2015; Ullah et al., 2018). ABRE (CGTGG/TC) is generally found in the promoters of ABA-inducible genes (Nakashima & Yamaguchi-Shinozaki, 2013). Our analysis predicted the presence of the ABRE motif in 15 drought-related *PtrAHL* genes. The qRT-PCR showed a significant change in the expression levels of 13 *PtrAHL* genes, except for *PtrAHL14* in roots and *PtrAHL36* in leaves, compared with control, indicating their role in the ABA signaling pathway. Previous studies have demonstrate that ABA in the guard cells induces stomatal closure during drought (Lim et al., 2015). Moreover, signals initiated in the roots under drought stress are transmitted quickly to the shoot and leaves, which lead to stomatal closure (Malcheska et al., 2017). We found that many drought-related *PtrAHL* genes such as *PtrAHL9, 20, 22, 27, 29,* and *34*, were induced by ABA treatment. These genes were significantly upregulated in roots and leaves under drought stress and ABA treatment, indicating their role in ABA-dependent signaling in response to drought stress. *PtrAHL27* was remarkably up-regulated in both roots and leaves at all time points under drought stress and ABA treatment, which indicates its critical role in response to drought stress and ABA treatment in *P. trichocarpa*.

### The predicted interaction network of *PtrAHL* proteins

Protein interaction network provides an understanding of orthologous proteins’ roles in biological processes (Hao et al., 2016). We used STRING to predict the protein interaction network of AHLs of *P. trichocarpa*. Our constructed networks revealed that, PtrAHL2/PtrAHL17 and PtrAHL12/PtrAHL35, which are homologs of the Type-II subfamily, shared a similar protein interaction network. These four proteins interact with HDA904, the *Arabidopsis* gene homologous to HDA9. Increasing evidence showed that *HDA9* acts in association with an ABA-related transcription factor to repress gene expression through histone deacetylation during drought stress (Baek et al., 2020). Moreover, *had9* mutant was insensitive to ABA and hypersensitive to drought stress by regulates the chromatin modification of genes responsible for regulation of both the ABA-signaling and ABA catabolosm pathways in response to ABA and drought stress (Khan et al., 2020). Research also has demonstrated that AtAHL22 interacts with HDAC, including HDA9, and influences the physiological processes such as flowering time (Yun et al., 2012). In our experiments, *PtrAHL12* and *PtrAHL17* were induced by ABA treatment and drought treatment, respectively. Therefore, we speculate that PtrAHL12 and 17 may interact with HDA904 and participate in drought stress response via a ABA-dependent pathway in *P. trichocarpa*.

## Conclusions

In summary, our study for the first time reports 37 AHLs in *P. trichocarpa*. The comprehensive analysis of the phylogeny, conserved motifs, and protein interaction network provides useful information about AHLs. Furthermore, a systematic approach was used to understand *PtrAHL* genes’ roles in response to drought stress and ABA treatment based on promoter *cis*-element and qRT-PCR analyses. Our study’s findings will help identify candidate *AHL* genes and provide a theoretical basis for further research on the functions of *AHL* genes under ABA treatment and drought stress in *P. trichocarpa*.

##  Supplemental Information

10.7717/peerj.10932/supp-1 Table S1 The primers for qRT-PCR

10.7717/peerj.10932/supp-2 Table S2 The value of tissue specific expression pattern in *PtrAHLs*

10.7717/peerj.10932/supp-3 Table S3 The datailed statistical analysis for qRT-PCR

10.7717/peerj.10932/supp-4 Table S4 The phylogenetic distance of AHLs between multiple lineages

10.7717/peerj.10932/supp-5 Table S5 Detailed information about 15 conserved motifs

10.7717/peerj.10932/supp-6 Table S6 The value of Ka, Ks and Ka/Ks as well as divergent date and duplicate type within 16 paralogous paris

10.7717/peerj.10932/supp-7 Table S7 The detail GO information of AHL genes in *P. trichocarpa.*

10.7717/peerj.10932/supp-8 Table S8 The raw data for qRT-PCR

10.7717/peerj.10932/supp-9 Table S9 Chromosomal location data

## Additional Information and Declarations

### Data Availability

The following information was supplied regarding data availability: Raw measurements are available in the Supplemental Files.
