# Supplementary material for: Comprehensive analysis of AHL gene family and their expression under drought stress and ABA treatment in Populus trichocarpa
Source: PeerJ. 2021 Feb 17;9:e10932. doi: 10.7717/peerj.10932 (PMC7896510; doi:10.7717/peerj.10932)
Supplement: Table S1 [file peerj-09-10932-s001.doc]

Supplementary Table S1. The primers for qRT-PCR.

| **Primer name** | **Primer sequence (5’→3’)** |
| --- | --- |
| PtrActin.F | TGTTGCCCTTGACTATGAGCAGGA |
| PtrActin.R | ACGGAATCTCTCAGCTCCAATGGT |
| PtrAHL8.F | ATAATAAAAAATAGTGTTTG |
| PtrAHL8.R | CACCCCTGTCTCCGACCCCG |
| PtrAHL9.F | ATGGCTGGAGCTGCAGACCT |
| PtrAHL9.R | CCACGAGGACGCCTGATCAT |
| PtrAHL12.F | TCTGGACCGGTGGTGATCAT |
| PtrAHL12.R | ATTCTGAGGCAACCCTTGAA |
| PtrAHL14.F | TTACTGTACTACTTCAGTTG |
| PtrAHL14.R | CAACAATATCACTCTCATCA |
| PtrAHL16.F | AAGCTCAAGTTTTTATTTAT |
| PtrAHL16.R | CAAATTTTCATTGAGAATTA |
| PtrAHL17.F | ATGGCCATTCTCTTCCACCT |
| PtrAHL17.R | GAGCTCTTTGCCTTCGCTAT |
| PtrAHL20.F | TACTGGTGTCAATAATGAAT |
| PtrAHL20.R | CCAAGCTTATTTAATTCTTG |
| PtrAHL21.F | CGTGTATATTTCTAGTGGGC |
| PtrAHL21.R | TAAGGAGAATAAAAAACACG |
| PtrAHL22.F | CTTCCTTGTAAAAGCACTTC |
| PtrAHL22.R | TATCATGTCAGAAAATAGGT |
| PtrAHL23.F | AAGAAATTTATGATGAATTT |
| PtrAHL23.R | ACAAGAATCCAGTAAAAATA |
| PtrAHL27.F | CTGACTCCGTCCATTTCCCT |
| PtrAHL27.R | TGATGGAGAGTCTGCAGTTA |
| PtrAHL29.F | CTTCCAATCTCTAACTACAA |
| PtrAHL29.R | CCTCATCTCCGGTATCTCTA |
| PtrAHL31.F | ACAGCATTTCTGCTGATTCA |
| PtrAHL31.R | CAACGCTACAAAACACAAGA |
| PtrAHL34.F | CTAATCTAAAGTAGGCTTCT |
| PtrAHL34.R | CCAAACAGTTTTTTTCATTA |
| PtrAHL36.F | GCACAGCCAGAAGTATTCAT |
| PtrAHL36.R | AATAAAAAAGCTTTATCCAA |
