# Supplementary material for: Comprehensive analysis of AHL gene family and their expression under drought stress and ABA treatment in Populus trichocarpa
Source: PeerJ. 2021 Feb 17;9:e10932. doi: 10.7717/peerj.10932 (PMC7896510; doi:10.7717/peerj.10932)
Supplement: Table S5 [file peerj-09-10932-s005.doc]

Supplementary Table S2. Detailed information about 15 conserved motifs.

| **Motif** | **Motif Consensus** |
| --- | --- |
| motif 1 | FTPHVIEVAAGEDIVEKVASFSQRRPRGVCILSGSGTVSNV |
| motif 2 | GGTVTYHGRFEILSLSGSFLP |
| motif 3 | LSVSLAGPQGQVVGGSVAGLL |
| motif 4 | RRPRGRPPGSKNKPKPPIIITRDSPNA |
| motif 5 | VVVMAASFANAAYERLPLEED |
| motif 6 | PVKKKRGRPRKYGPDGAVALA |
| motif 7 | TLRQPASS |
| motif 8 | ASPVQVVVGSF |
| motif 9 | GGQQQNPQQHQVMAEPGALPFFGLPPNLLNNVQLPAEA |
| motif 10 | YHHQDHHRHQFHHQQQQNSEDEQSGN |
| motif 11 | QDMRNKHTDIBLTRG |
| motif 12 | DYNGGKPGKVWPGIYGKPKYKKHGMZNLGLWAANN |
| motif 13 | EDGGTRSRSGG |
| motif 14 | LAGNQQEQKPKKQKIDSJPAVFPPA |
| motif 15 | KNKSVKREEDEDDSDSTNVEN |
