# Supplementary material for: Comprehensive analysis of AHL gene family and their expression under drought stress and ABA treatment in Populus trichocarpa
Source: PeerJ. 2021 Feb 17;9:e10932. doi: 10.7717/peerj.10932 (PMC7896510; doi:10.7717/peerj.10932)
Supplement: Table S6 [file peerj-09-10932-s006.doc]

Supplementary Table S3. The value of Ka, Ks and Ka/Ks as well as divergent date and duplicate type within 16 paralogous paris.

| **Paralogous pairs** | **Ks** | **Ka** | **Ka/Ks** | **Divergent** date (Mya) | **Duplicate type** |
| --- | --- | --- | --- | --- | --- |
| PtrAHL1-PtrAHL18 | 0.2107 | 0.0778 | 0.3692 | 6.38 | Segmental |
| PtrAHL2-PtrAHL17 | 0.3101 | 0.0166 | 0.0535 | 1.36 | Segmental |
| PtrAHL4-PtrAHL15 | 1.379 | 0.7152 | 0.5186 | 58.62 | Segmental |
| PtrAHL5-PtrAHL14 | 0.2363 | 0.0702 | 0.2971 | 5.75 | Segmental |
| PtrAHL6-PtrAHL27 | 0.2303 | 0.0819 | 0.3556 | 6.71 | Segmental |
| PtrAHL7-PtrAHL24 | 0.0857 | 0.1277 | 1.4901 | 10.47 | Segmental |
| PtrAHL8-PtrAHL23 | 0.1864 | 0.0819 | 0.4394 | 6.71 | Segmental |
| PtrAHL9-PtrAHL22 | 0.3629 | 0.0516 | 0.1422 | 4.23 | Segmental |
| PtrAHL10-PtrAHL21 | 0.4614 | 0.0589 | 0.1277 | 4.83 | Segmental |
| PtrAHL11-PtrAHL34 | 0.2366 | 0.0269 | 0.1137 | 2.20 | Segmental |
| PtrAHL12-PtrAHL35 | 0.2451 | 0.0447 | 0.1824 | 3.66 | Segmental |
| PtrAHL13-PtrAHL36 | 0.2505 | 0.0669 | 0.2671 | 5.48 | Segmental |
| PtrAHL19-PtrAHL28 | 0.2401 | 0.0862 | 0.3590 | 7.07 | Segmental |
| PtrAHL25-PtrAHL30 | 0.2449 | 0.0472 | 0.1927 | 3.87 | Segmental |
| PtrAHL26-PtrAHL29 | 0.3215 | 0.0359 | 0.1117 | 2.94 | Segmental |
| PtrAHL32-PtrAHL37 | 0.2924 | 0.0878 | 0.3003 | 7.20 | Segmental |
